# Lipid-Based Nanoparticles in the Clinic and Clinical Trials: From Cancer Nanomedicine to COVID-19 Vaccines

**DOI:** 10.3390/vaccines9040359

**Published:** 2021-04-08

**Authors:** Thai Thanh Hoang Thi, Estelle J. A. Suys, Jung Seok Lee, Dai Hai Nguyen, Ki Dong Park, Nghia P. Truong

**Affiliations:** 1 Biomaterials and Nanotechnology Research Group, Faculty of Applied Sciences, Ton Duc Thang University, Ho Chi Minh City 700000, Vietnam; hoangthithaithanh@tdtu.edu.vn; 2 Monash Institute of Pharmaceutical Sciences, Monash University, Parkville, VIC 3052, Australia; estelle.suys@monash.edu; 3 Biomedical Engineering, Malone Engineering Center 402A, Yale University, 55 Prospect St., New Haven, CT 06511, USA; jungseok.lee78@gmail.com; 4 Vietnam Academy of Science and Technology, Graduate University of Science and Technology, Hanoi 100000, Vietnam; nguyendaihai@iams.vast.vn; 5 Institute of Applied Materials Science, Vietnam Academy of Science and Technology, 01 TL29 District 12, Ho Chi Minh City 700000, Vietnam; 6 Department of Molecular Science and Technology, Ajou University, Suwon 16499, Korea; kdp@ajou.ac.kr

**Keywords:** lipid nanoparticles, liposomes, vaccines, immunotherapy, COVID-19

## Abstract

COVID-19 vaccines have been developed with unprecedented speed which would not have been possible without decades of fundamental research on delivery nanotechnology. Lipid-based nanoparticles have played a pivotal role in the successes of COVID-19 vaccines and many other nanomedicines, such as Doxil^®^ and Onpattro^®^, and have therefore been considered as the frontrunner in nanoscale drug delivery systems. In this review, we aim to highlight the progress in the development of these lipid nanoparticles for various applications, ranging from cancer nanomedicines to COVID-19 vaccines. The lipid-based nanoparticles discussed in this review are liposomes, niosomes, transfersomes, solid lipid nanoparticles, and nanostructured lipid carriers. We particularly focus on the innovations that have obtained regulatory approval or that are in clinical trials. We also discuss the physicochemical properties required for specific applications, highlight the differences in requirements for the delivery of different cargos, and introduce current challenges that need further development. This review serves as a useful guideline for designing new lipid nanoparticles for both preventative and therapeutic vaccines including immunotherapies.

## 1. Introduction

**Figure 1:**
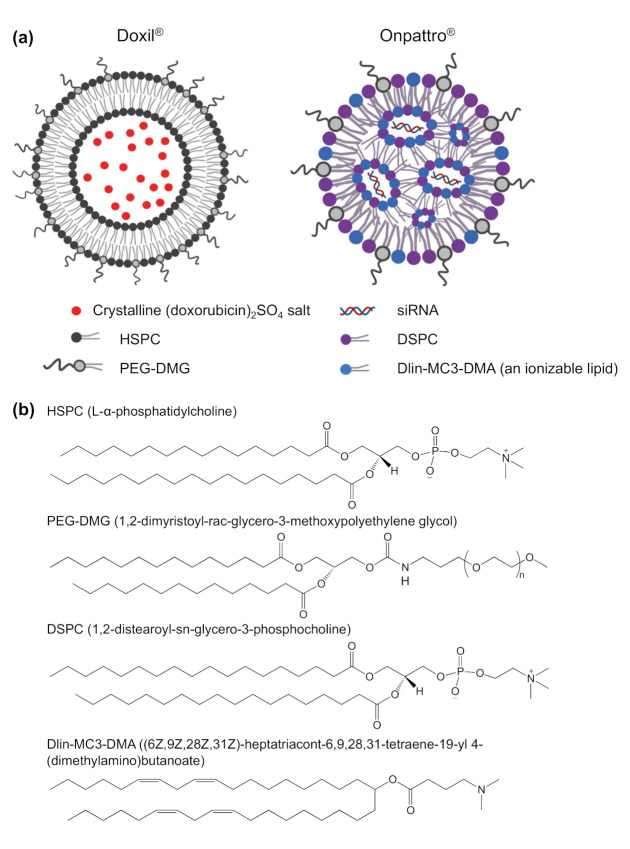
(**a**) Structure of FDA approved Doxil^®^ and Onpattro^®^ (patisiran) nanoparticles—the first FDA approved liposome and lipid nanoparticle, Created in BioRender.com; (**b**) chemical structure of the lipids inDoxil^®^ and Onpattro^®^.

Nanomedicine is the convergence of nanotechnology, pharmaceutical, and biomedical sciences and has developed rapidly with the design of new nanoformulations for therapeutic purposes, imaging agents and theragnostic applications. Nanoformulation was defined by the Food and Drug Administration (FDA) that is the products in combination with nanoparticles ranging from 1–100 nanometers (nm); or other formulations outside of this range showing dimension-dependent properties [1]. These formulations exhibit many advantages over free drug molecules, possessing an enhanced solubility and improved pharmacokinetics, efficacy, and minimal toxicity [1]. More than 50 nanopharmaceuticals have made it to the market consisting of diverse nanoformulations, with lipid nanoparticles being the frontrunner [1,2,3,4]. Lipid nanoparticles are multicomponent lipid systems typically containing a phospholipid, an ionizable lipid, cholesterol, and a PEGylated lipid [5]. The traditional type of lipid nanoparticles is liposomes which was first described in 1961 by the British haematologist, Alec D Bangham [6]. Liposomes were observed under the electron microscope when adding negative stain to dry phospholipids that assembled into spherical shape through a lipid bilayer. Later, in 1980, the first targeted liposomes, modified by active targeting ligands, were developed and led to significantly improve liposome capacity by increasing accumulation at the target tissues/organs/cells without releasing the drug to other sites [7]. As a result, the overall efficacy of these liposomes is improved compared to conventional liposomes. Though liposomes have been explored for 30 years as an effective carrier for a variety of drug molecules, it was only in the 1990s that the first Food and Drug Administration (FDA) approval came. This milestone was reached by Doxil^®^, a stealth liposome encapsulating doxorubicin (Figure 1a) and used for the clinical treatment of ovarian and metastatic breast cancer as well as various forms of myeloma [1]. Because of the encapsulation of doxorubicin inside PEGylated liposomes, the side effects of free doxorubicin, including chronic cardiomyopathy and congestive heart failure, were significantly mitigated [8]. In addition, the PEGylation of liposomes in Doxil^®^ supported prolonging the circulation time of this formula after administration [8]. Thereafter, the passive accumulation into tumours was achieved. Overall, Doxil^®^ has significant cardiotoxicity reduction and high anticancer ability compared to free doxorubicin due to the enhanced permeability and retention effect [9].

The start of the 21st century marks the paradigm-shifting development of multi-component formulations for delivering oligonucleotides for gene therapies [2]. These oligonucleotides are macromolecules that exhibit higher therapeutic indexes than conventional chemotherapeutics, especially when the formulation is tailored to reach specific tissues [10]. The main challenge gene delivery facing is the instability of naked nucleic acids in physiological media [11]. The development of suitable formulations that guarantee sufficient in vivo stability as well as tissue targeting ability has therefore been crucial. This advancement was achieved in 2018, with the FDA approval of Onpattro^®^ ((Alnylam Pharmaceuticals, Inc., Cambridge, MA, USA) and Sanofi Genzyme (Cambridge, MA, USA)), consisting of siRNA encapsulated in lipid nano-particles (LNPs) (Figure 1a) for the treatment polyneuropathy in people with hereditary transthyretin-mediated amyloidosis [12,13,14,15]. This LNP is made of (6Z,9Z,28Z,31Z)-heptatriaconta-6,9,28,31-tetraen-19-yl-4-(dimethylamino)-butanoate (DLin-MC3-DMA) lipid, disterarolyphosphatidychloline (DSPC), cholesterol and a PEG-lipid (PEG-DMG) that directs the particle in vivo towards the liver hepatocytes [15,16,17]. The ionizable cationic lipid in the LNP complexes with the nucleic acids in acidic media (pH~4) by electrostatic interaction. At physiological pH of 7.4, this formulation becomes neutrally charged and thereby more stealth which dampens the interaction with blood components. Upon internalization of these LNPs in cells, these structural lipids become positively charged, which promotes complexation with the negatively charged endo/lysosomal membrane. This interaction with cellular compartments then disrupts and releases the nucleic acid in the cytosol, where they can exert their effect. Studies have suggested that the structure and pKa of the ionizable lipids play a crucial role in the delivery efficiency of the cargo to the target cells [5,18,19]. For example, Dlin-MC3-DMA, the ionizable lipid in Onpattro (Figure 1b), with a pKa of 6.44, has a 10-fold higher potency than (2,2-dilinoleyl-4-(2- dimethylaminoethyl)-[1,3]-dioxolane, DLin-KC2-DMA) with a pKa of 6.7 [20,21]. However, it should be noted that subtle differences in the lipid structure can induce variations in the structural packing of LNP’s, resulting in a different morphology and delivery profile. To improve the particle stability, DSPC, with two saturated acyl chains and a large head group, is incorporated into the formulation (Figure 1b). These lipids produce a cylindrical geometry and play the role of helper lipids in the LNP to maintain the outer layer structure and increase the Onpattro’s formation [22]. The PEG-DMG lipids on the particle surface (Figure 1a,b) prevent particle aggregation due to stealth properties and prolong the circulation time in vivo. Altogether, this LNP system has a solid core structure, a low surface charge at physiological media, and low immunogenicity [16]. This technology has sparked interest in further developing genetic therapies and novel delivery systems. Lipid-based nanoparticles are a particularly promising vehicle [11,23,24] for gene delivery with their higher biocompatibility than polymeric and inorganic nanoparticles, their inherent penetrating ability, their biodegradability, structural flexibility, and low immunogenicity [25,26]. These nanostructures can also be produced rapidly at large scale, which is a major advantage when moving into clinical trials and commercial applications [25]. 

Since the success of Doxil, Onpattro, and various other LNPs in clinical trials, a myriad of studies has been conducted to translate R&D efforts into commercial products. This review describes the various FDA-approved lipid-based nanoparticles to provide our perspectives on the opportunities and challenges of future developments. Particularly, we first aim to provide an overview of lipid-based nanoparticles and their structure/property relationship. Next, we survey lipid nanoparticles that have been successfully used for cancer drug delivery. We also discuss the more challenging delivery of genetic materials and, finally, its application in preventative vaccines against viruses as well as therapeutic vaccines against cancers, including immunotherapies.

## 2. Lipid-Based Nanoparticles

### 2.1. Overview of Lipid-Based Nanoparticles

**Figure 2:**
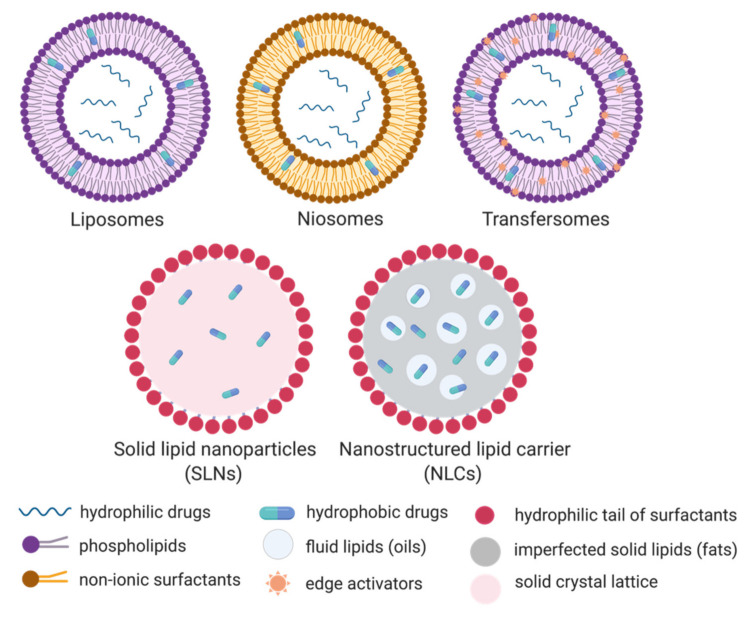
Schematic representation of the five categories of lipid-based nanoparticles: Liposomes, niosomes, transfersomes, solid lipid nanoparticles (SLNs) and the nanostructured lipid carriers (NLCs). Created in BioRender.com.

Lipid-based nanoparticles are classified into 5 categories depending on the fabrication method and on the physicochemical properties of the formulations. These are liposomes, niosomes, transfersomes, solid lipid nanoparticles (SLNs) and nanostructured lipid carriers (NLCs) [25]. The main properties of each type of particle are described in Figure 2 and Table 1. In brief, liposomes, structured by phospholipids and cholesterol, have great drug protection and targeting abilities [27]. These properties lead to liposomes having reduced toxicity with improved efficacy over free drug in the systemic circulation [6]. The application of liposomes in dermal delivery is limited due to their lack of penetrating ability in the stratum corneum. Additional drawbacks include the poor encapsulation of hydrophilic drugs and the weak storage stability due to drug leakage in the media [28]. An alternative to liposomes is niosomes, formed by nonionic surfactants and cholesterol in aqueous conditions, and provide better stability and longer shelf life than their liposomal counterpart [27,29]. Moreover, their neutral shell exhibits compatibility when compared to positively charged liposomes. Despite their advantageous properties, niosomes also suffer from drug leakage and particle aggregation due to a lack of ionic repulsion, which has been a barrier to grant FDA approvals [29,30]. Transfersomes are elastic or deformable nanoparticles composed of phospholipids, edge activators (EA) and cholesterol [28]. The addition of an EA enhances their flexibility which promotes higher tissue permeation. Transfersomes show the highest penetration capability and entrapment efficiency for lipophilic molecules [28]. Their oxidative degradation and high material cost remain a challenge to produce this type of particle in large quantities. All these lipophilic bi/multi-layer nanoparticles with hydrophilic core are able to encapsulate both hydrophilic and hydrophobic drugs without chemical intervention. Hydrophobic drugs are preferentially sandwiched in the external bilayer, while hydrophilic molecules are incorporated in the aqueous cavity (Figure 2). These particles are therefore considered an effective carrier for a wide spectrum of drugs, with sufficient protection, sustainable drug release, and improved bioavailability.

The drawbacks related to their limited stability, toxicity, low loading capacity, and convoluted manufacturing has sparked the interest of researchers to develop a new class of particles became [25,29], mainly aiming at improving the encapsulation efficiency [12]. Solid lipid nanoparticles (SLNs, Figure 2) are made of solid fats and surfactants to form a spherical nanoparticle with a solid lipid core and a monolayer shell [33,34]. They exhibit greater encapsulation efficiency for hydrophobic drugs than liposomes since they lack an aqueous core. This rigid core also improved the stability of SLNs compared to liposomes and polymeric nanoparticles. Indeed, SLNs in aqueous media could be stored for at least 3 years [35]. The production of SLNs does not require organic solvents, which eliminates the risk of toxicity caused by solvent residues. Additionally, the large-scale production and excellent reproducibility of SLNs are important properties for downstream commercial and clinical applications [29]. Following on SLNs, nanostructured lipid carriers (NLCs) have been developed to enhance drug encapsulation and prevent drug leakage. NLCs are composed of an unstructured lipid interior and a monolayer surfactant periphery (Figure 2). The core is made of a mixture of solid and liquid lipids that form an imperfect crystal interior to increase the drug loading, while SLNs are saturating the drug loading due to a solid crystal lattice. In addition, this liquid phase in NLC inhibits drug release during storage [36].

Moreover, lipid-polymer hybrid nanoparticles have been studied to create synergies between lipid-based nanoparticles and polymeric particles. Indeed, Zhang et al. made a comparison between lipid-based nanoparticles, polymeric nanoparticles and the hybrid nanoparticles loaded with cisplatin, where the in vivo data suggests that cisplatin-loaded lipid-polymer hybrid nanoparticles inhibited the ovarian carcinoma most effectively [37]. Additionally, these hybrid particles have also been considered in gene delivery applications [24,38]. By optimizing the properties of lipid-based nanoparticles, the safety, encapsulating capacity, stability, pharmacokinetics, bio-distribution, and therapeutic benefit can be controlled as a result [39].

### 2.2. The Properties of Lipid-Based Nanoparticles Governing Their Efficiencies

For the delivery of oligonucleotides, nanoparticles need to encapsulate sufficient amounts of nucleic acid and have specific tissues targeting properties [12,13]. Thus, optimization of lipid-based nanoparticle is key for tailoring the delivery to the site of action [39]. Structural determinants, such as the particle size, surface charge, PEGylation, and surface modification by targeting ligands have shown to be critical elements in governing the delivery efficiency of these nanoparticles [13,24,39].

Small size nanoparticles have been shown to facilitate transport in blood and lymphatic capillaries as well as uptake in tissues [13,40]. More specifically, nanoparticles <10 nm are optimal for diffusion into blood capillaries, whilst particles of 10–100 nm are favorably uptaken into lymphatic capillaries by convection. When the dimensions are 100–200 nm their ability to diffuse rapidly is reduced [40]. A study by Oussoren et al. demonstrated that 40 nm liposomes had higher lymphatic uptake than larger particles after subcutaneous injection [41]. Specifically, 76% of the injected dose of these 40 nm liposomes was taken by the lymph nodes, while larger liposomes remained at the site of subcutaneous injection [41]. For liver targeting, after systemic administration, only particles <100 nm, were able to diffuse through the liver fenestrae to reach hepatocytes and hepatic stellate cells [42,43]. These results indicate that small-sized liposomes enhance lymph node transitivity, and crucially penetrate into liver fenestrae for targeting hepatocytes. For large-sized liposomes (>150 nm), these nanoparticles were taken up by the antigen-presenting cells (APCs) at an injection site, then were carried to the lymph nodes [44]. It was demonstrated that they gained a higher cell affinity within the lymph nodes [44].

**Figure 3:**
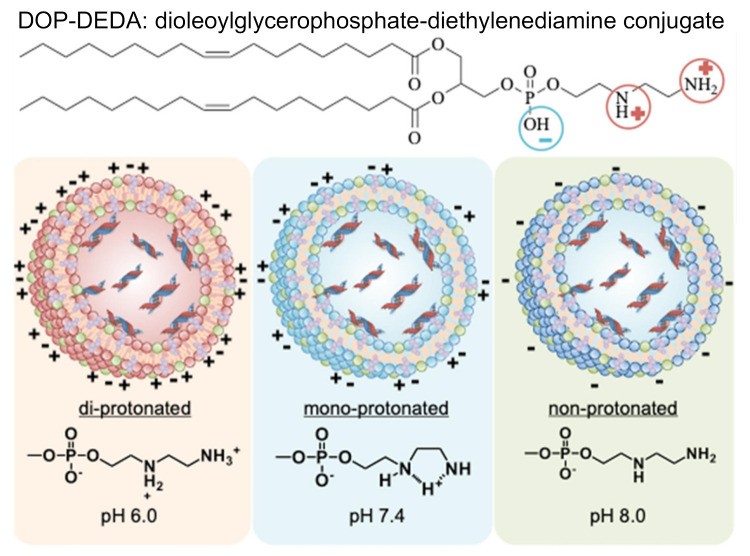
Impact of pH on the protonation and structure of charge-reversible lipid-based nanoparticles encapsulating siRNA. These lipid nanoparticles become positive charge at pH of 6.0, neutral at pH of 7.4 and a negative charge at pH of 8.0 gained due to an ionizable lipid of di-oleoylglycerophosphate-diethylenediamine conjugate (DOP-DEDA). Used with permission from [48].

In the case of the charge effect of lipid-based nanoparticles, this was a hard aspect to reach a general conclusion. Mai et al. reported that an anionic and cationic liposome exhibited significantly higher association with B cells than uncharged liposome within microvascular network [45]. This phenomenon was explained by the similar complemental proteins of anionic, cationic liposome shells with those being able to interact with B cells. Cationic liposomes showed the highest levels of interaction and internalization by B cell receptor. Cationic liposomes were in situ decorated with opsonins in blood which is recognized by human immune cells. Anionic liposomes were found to mostly attach on the surface of B cells. This finding suggests that charged liposomes might be suitable for vaccine application, while neutral particles might be more suitable for the delivery of chemotherapies [45]. Nakamura et al. found that 30 nm negatively charged LNPs were able to target the lymph node more effectively than positively charged and neutral particles [40]. The results showed that 20–30% of the B220+ cells in the LN were DiD-labelled from the neutral and positively charged LNPs, whereas almost 80% of the B220+ cells were labelled with the negatively charged LNPs [40]. Retention of LNPs is at the site of injection was shown to be more pronounced with cationic particles compared to neutral and anionic particles [13,46]. This effect is due to the high electrostatic interaction between the cationic LNPs and negatively charged tissues. Additionally, cationic LNPs have been reported to bind nonspecifically with plasma proteins and have been linked to higher immunogenicity [47]. Taken together, positively charged LNPs have strong cellular affinity but have limited efficacy [47], while negatively charged LNPs show to be transported to lymph node effectively. To take advantage of this charge aspect, charge-reversible LNPs have been designed by Hirai et al. to achieve the best of both worlds in gene delivery [48]. These LNPs, composed of dipalmitoylphosphatidylcholine (DPPC), cholesterol, and dioleoylglycerophosphate-diethylenediamine conjugate (DOP-DEDA) are positively charged at pH of 6.0, neutral at pH of 7.4 and negatively charged at pH of 8.0. This system is neutral in the bloodstream to minimize degradation by plasma proteins and protect the encapsulated cargo. When circulating in the bloodstream, these DOP-DEDA-LNP vehicles bind to apolipoproteins (e.g., apoE3) at their hydrophobic lipid regions, which promotes their uptake by cancerous cells via both clathrin and caveola-mediated endocytosis pathways. In the endo-lysosomal compartment, the pH is low, which leads to DOP-DEDA-lipid nanoparticles being positively charged for enhanced cytosolic penetration in target cells (Figure 3) [48]. These LNPs with pH-dependent charge-invert properties are thought to be a safe and effective vector to induce RNAi-mediated gene-silencing [48]. Another strong determinant for the potency of LNPs is the lipid pKa. A series of studies have shown that a pKa of 6.4 is optimal for maximizing the transfection for siRNA-LNPs [49,50]. This optimal pKa is also changed in the case of mRNA, with an optimal range of 6.6–6.8 [49].

**Figure 4:**
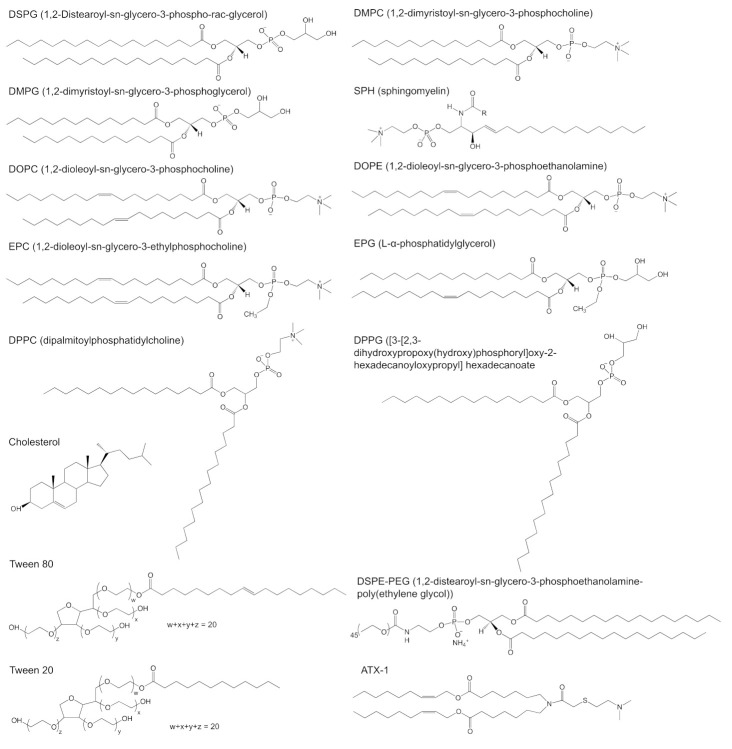
Chemical structure of targeting lipids? DSPG (1,2-distearoyl-sn-glycero-3-phospho-rac-glycerol), DMPC (1,2-dimyristoyl-sn-glycero-3-phosphocholine), DMPG (1,2-dimyristoyl-sn-glycero-3-phosphoglycerol), SPH (sphingomyelin), DOPC (1,2-dioleoyl-sn-glycero-3-phosphocholine), DOPE (1,2-dioleoyl-sn-glycero-3-phosphoethanolamine), EPC (1,2-dioleoyl-sn-glycero-3-ethylphosphocholine), EPG (L-α-phosphatidylglycerol), DPPC (dipalmitoylphosphatidylcholine), DPPG ([3-[2,3-dihydroxypropoxy(hydroxy)phosphoryl]oxy-2-hexadecanoyloxypropyl]hexadecanoate, cholesterol, Tween 80 (2-[2-[3,5-bis(2-hydroxyethoxy)oxolan-2-yl]-2-(2-hydroxyethoxy)ethoxy]ethyl (E)-octadec-9-enoate), Tween 20 (2-[2-[3,4-bis(2-hydroxyethoxy)oxolan-2-yl]-2-(2-hydroxyethoxy)ethoxy]ethyl dodecanoate), ATX-1 (one of the LUNAR lipids of Arturus Therapeutics, Inc., San Diego, CA, USA) and DSPE-PEG (1,2-distearoyl-sn-glycero-3-phosphoethanolamine-poly(ethylene glycol)).

As a strategy for improving the targeting capacity, PEGylation of lipid-based nanoparticles has gained much interest in an attempt to reduce the clearance of particles from the bloodstream, and thereby increasing their retention and uptake in tissues into targeting tissues/organs. Studies of Moghimi have revealed that the PEGs modifying anionic liposomes were able to achieve better clearance at the site of subcutaneous administration and higher retention in the lymph nodes compared with bare liposomes. In the case of PEG length, the shorter PEG chains gained lower clearance but higher retention in the lymph nodes compared with the longer PEG modified liposomes [51]. Structurally, reports have shown that using a linear or branched PEG chain can significantly dictate the targeting behaviour and transfection ability of LNPs. Truong group synthesized three LNPs with either Tween 80 (2-[2-[3,5-bis(2-hydroxyethoxy)oxolan-2-yl]-2-(2-hydroxyethoxy)ethoxy]ethyl (E)-octadec-9-enoate), Tween 20 (2-[2-[3,4-bis(2-hydroxyethoxy)oxolan-2-yl]-2-(2-hydroxyethoxy)ethoxy]ethyl dodecanoate) and 1,2-distearoyl-sn-glycero-3-phosphoethanolamine-poly(ethylene glycol) (DSPE-PEG) to encapsulate pDNA and investigate their targeting ability, stability and extent of transfection [52]. Tween 20 and Tween 80, both non-ionic surfactants are made of saturated carbon tails with similar branched PEG architecture, compared to the linear structure of DSPE-PEG (Figure 4). Despite these structural differences, the LNPs exhibited similar stability over a 3-week period. In vivo transfection studies demonstrated that particles with the shorter Tween 20 were able to target the lymph nodes more efficiently. The longer Tween 80, on the other hand, formed LNPs that targeted the spleen but with lower efficiency. LNPs with the linear DSPE-PEG showed predominantly localized transfection at the injection site. Altogether, this study demonstrated that PEGylation of LNPs with branched PEG is a viable approach to target organs with effective transfection selectively [52]. It is hypothesized that, aside from the molecular weight and structure, the PEG density on the particle is also an important factor. Across the three types of LNPs, PEGylation of positively charged lipid nanoparticles showed much difference than bare ones [52]. PEGylation prevented positively charged lipid nanoparticles from high retention at the site of administration. Whilst PEGylation has been proven useful to prevent premature clearance of particle from the systemic circulation, the production of anti-PEG antibodies has emerged as detrimental co-lateral damage. Recently, efforts have been directed towards alternative strategies with biocompatible polymers to prevent the production of anti-PEG antibodies after the first dose that would otherwise lead to a loss of therapeutic efficacy with potential for adverse effects upon subsequent doses [53]. Concurrently, Chen et al. found that incorporation of 4 mol% dexamethasones in lipid-based nanoparticles was able to suppress the immune responses and antibody production after injection [26], which might be promising for the development of pegylated lipid nanocarriers.

Aside from relying on prolonged circulation and passive cellular uptake of nanoparticles, formulation scientists have realized that, by conjugation of a receptor ligand to the particle surface, uptake into target cells could be enhanced. Modification of lipid-based nanoparticles with targeting ligands is a key strategy for efficiently targeting delivery systems into the lymph nodes or other desired tissues/cells. For example, Vu et al. functionalized liposome surface with Hemagglutinin Antigen (HA) to improve antibody production efficiency [54]. This work showed that HA-functionalized liposome could cross the barrier cells in the lymph node and enhance germinal center formation and follicular helper T Cell Immunity. Monoclonal antibodies (mAbs) are also a promising avenue for conjugation onto nanoparticle surfaces via covalent bonds for leukocyte targeting [39]. Veiga et al. conjugated anti-Ly6C antibodies on lipid-based nanoparticles loaded with siRNA to form the ASSET platform (Anchored Secondary scFv Enabling Targeting) [55]. This formulation was able to selectively target inflammatory leukocytes in vivo, as shown in other studies [56,57]. Similarly, solid tumors often display expressed higher levels of p32 than non-cancerous tissues. To take advantage of this, linTT1 (AKRGARSTA) and LyP-1 peptides have been developed, that can bind to p32 with strong affinity [58]. These peptides were therefore utilized as targeting ligand for tumor homing. Indeed, Hunt et al. used linTT1 as targeting ligand in peritoneal carcinomatosis, and found that these nanoparticles could be absorbed to a greater extent than non-targeted particles in peritoneal tumors in mice [59]. Säälik et al. also demonstrated a similar result using linTT1 as a targeting ligand for tumor homing in vitro and in vivo [58]. For the treatment of neurological disorder, targeting the nervous system is a requisite. Kuo et al. designed a formulation with amphiphilic solid lipid nanoparticles decorated with the Ln5-P4 (PPFLMLLKGSTR) peptide (Ln5-P4-ASLNs) for co-delivery of nerve growth factor (NGF) and retinoic acid (RA) [60]. Ln5-P4 bound the α_3_β_1_ integrin and supported cell adhesion and spreading to guide the differentiation of induced pluripotent stem cells toward neurons. This formulation proved to increase the survival rate of induced pluripotent stem cells and the generation of mature neurons as a potential treatment of neurodegenerative diseases and nerve injury in regeneration medicine. In other studies, selectivity for neuronal cells was improved by attachment of the rabies virus glycoprotein (RVG) peptide, a fragment of from the rabies virus glycoprotein, on the surface of siRNA-loaded exosomes [61]. This formulation showed positive effects in a mouse model of Alzheimer’s disease. To enhance the cellular uptake in dendritic cells for enhanced immune response, lipid-based nanoparticles have been conjugated with cell-penetrating peptides such as R8 and GALA [62]. Many other ligands of dendritic cell receptors (for review see [63]) and of liver cells (for review see [17]) have been developed for targeting purposes and are listed below. Fusogenic peptides such as DOPE have also shown to improve membrane fusion, and thus cell uptake [64]. Transferrin, folic acid, and antibodies can be used as a ligand for lipid-based nanoparticles to target cancer cell receptors [65,66,67,68]. For the objective of improving siRNA transfection, NP3.47, an inhibitor of the Niemann-Pick type C-1 protein (NPC-1), was conjugated to the surface of the lipid-based nanoparticle. NP3.47 promoted the accumulation of siRNA-LNPs in late endosomes/lysosomes up to 3-fold higher than unmodified lipid-based nanoparticles [69]. Due to the increased trapping of NP3.47-LNP-siRNA systems in late endosomes, enhanced opportunities for endosomal escape can be gained from this work for the delivery of siRNA and other oligonucleotide [69].

In recent years, lipid-based nanoparticles have emerged as the most effective carrier for the delivery of cargo to target cells, which have translated into clinical success. The analytical characterization, the basic technological concepts and highlights have been reviewed extensively before [17,38,39,70]. The current review will focus on the translation of lipid-based nanoparticles into the clinic.

## 3. Lipid-Based Nanoparticles for Drug Delivery

For almost 30 years, liposomes have been blossoming in clinical applications. Twenty-one liposomal products have been approved, encapsulating different small molecule drugs. The clinical success of Doxil^®^ has given rise to the approval of many new nanodrugs by the FDA, such as Abelcet^®^, AmBisome^®^, DaunoXome^®^, Depocyt^®^, Inflexal V^®^, Myocet^®^, Visudyne^®^, DepoDur^®^, DepoCyt^®^, Marqibo^®^, Mepact^®^, Exparel^®^, Lipodox^®^, Onivyde^®^, Doxorubicin, Nocita^®^, Vyxeos^®^, Shingrix^®^, Lipoplatin^TM^, and Arikayce^®^ [1,2,6,71]. These formulations are not only used in oncology but also in fungal infections and pain management. Each liposome formulation is described in greater detail in Table 2, with their respective structural lipids shown in Figure 4. There are four different liposomal products with doxorubicin (Doxil^®^, Myocet^®^, Lipodox^®^, and Liposomal doxorubicin) that are indicated for breast neoplasms. Of these products, Myocet^®^ is a conventional formulation, while Doxil^®^, Lipodox^®^, and Doxorubicin are stealth, pegylated liposomes. Aside from these three stealth liposomes, there is only one other product, Onivyde^®^ that contains stealth properties by PEGylation. For anaesthetic applications, there are two different liposomes approved that encapsulate bupivacaine. For life-threatening fungal infections, there are also two liposomal formulations on the market that encapsulate amphotericin B. Marqibo^®^, a conventional liposome loaded with vincristine, was approved in 2009 for the treatment of acute lymphoblastic leukaemia and in 2012 for hematologic malignancy and solid tumor treatment. In addition, other drugs including daunorubicin, cytarabine, verteporfin, morphine, mifamurtide, irinotecan, cytarabine, and amikacin were formulated in liposomes. Among them, only Curosurf^®^ encapsulated surfactant protein B and C (SP-B and SP-C) in a conventional liposome for the treatment of respiratory distress syndrome (RDS) in premature infants. An atypical formulation is Vyxeos^®^, which has two drugs incorporated into the liposomes, thereby exploiting the synergistic effect of daunorubicin and cytarabine to treat acute myeloid leukaemia more effectively. In summary, within this period, there were 13 chemotherapeutics and one protein formulated in conventional and stealth liposomes successfully commercialized.

Aside from those formulations, there are many other liposomes indicated for chemotherapy in currently in clinical trials (Table 3). MBP-426^®^ of Mebiopharm Co., Ltd. (Tokyo, Japan) is an oxaliplatin-encapsulated transferrin-conjugated N-glutaryl phosphatidylethanolamine-liposome and is indicated for gastric, oesophageal and gastro-oesophageal adenocarcinoma. The phase I clinical trial (NCT00355888) of MBP-426^®^ was completed [7], with phase IIa/b starting (NCT00964080) for characterization of the safety profile in combination with leucovorin and fluorouracil. Mebiopharm Co., Ltd. has also developed other products including MBP-Y003, MBP-Y004, and MBP-Y005 in preclinical stages which are transferrin-conjugated liposomes loaded with methotrexate, docetaxel, and gemcitabine, respectively [73]. These four products are designed with a transferrin ligand for targeting receptors that are overexpressed in cancerous tissues.

ThermoDox^®^ of Celsion is a heat-sensitive liposome loaded with doxorubicin for the treatment of hepatocellular carcinoma. The thermosensitive lipid is able to change structure at 40–45 °C to release doxorubicin rapidly in the tumor through radiofrequency ablation. Although four commercialized liposomes loading doxorubicin were already launched into the market successfully, ThermoDox^®^ is a new product with advanced characteristics, that showed a 5-fold release in doxorubicin concentration at the tumor site when compared to Doxil^®^ [74]. Phase III clinical trials of ThermoDox^®^ in combination with standardized radiofrequency ablation (NCT02112656) have been completed [7,74].

MM-302 of Merrimack Pharmaceuticals, is a stealth liposome modified with antibodies targeting the human epidermal growth factor receptor 2 (HER2) and loaded with doxorubicin [75], which has applied for phase 1 clinical trial in 2011. MM-302 aimed at overcoming the limitations of doxorubicin related to cardiac toxicity and to the ineffective targeting of cancerous cells. MM-302 was assessed in combination with trastuzumab or trastuzumab plus cyclophosphamide to treat advanced HER2-positive breast cancer. The promising data of phase 1 clinical trials inspired MM-302 to move to phase 2. However, the efficacy results did not show significant benefit compared to comparator treatments, which led to Merrimack discontinuing further trials with MM-302 in 2016 [76]. 

SPI-77 developed by Sequus Pharmaceuticals (Johnson & Johnson) is a cisplatin-encapsulated stealth liposome, developed for the treatment of recurrent ovarian cancer [77] and stage IV non-small cell lung cancer (NSCLC) [78]. SPI-77 is hypothesized to mitigate the systemic toxicity of cisplatin and to achieve a high delivery capacity. However, due to lack of significant data, in phase 1 and phase 2 clinical trials, the manufacturer decided to halt further trials [77,78,79]. 

The liposome-encapsulated mitoxantrone (LEM) from INSYS Therapeutics Inc is made of lyophilized lipids mixed with mitoxantrone salt (under the commercial name of Novantrone). This formulation was generated to improve the safety and efficacy of free Novantrone. LEM entered phase 1 clinical trials in 2001 with an identifier of NCT00024492. Patients with advanced solid tumors (40 participants) were recruited for intravenous injection of LEM. The results from the blood pharmacokinetics and tumor observation were completed in 2004, but no results were posted.

OSI-211, a liposome encapsulating lurtotecan, was developed for treatment of recurrent small-cell lung cancer. OSI-211 was clinically tested (NCT00046787) by Astellas Pharma Inc. (Chuo City, Tokyo, Japan) The University of Pittsburgh and ALZA company developed S-CKD602 and completed phase 1 clinical trial (NCT00177281) to determine the maximum tolerated dose as well as the safety in patients with advanced tumors. S-CKD602 is a PEGylated liposome encapsulating CKD-602, a camptothecin analogue inhibiting topoisomerase I, with liposomal formulation consisting of N-(carbonyl-methoxypolyethylene glycol 2000)-DSPE and DSPC [80]. Another liposome named LEP-ETU also entered phase 1 clinical trials (NCT00080418). LEP-ETU is a liposome formed by DOPC, cholesterol and cardiolipin that encapsulates paclitaxel to treat ovarian, breast and lung cancers [81]. Topotecan Liposomes Injection (TLI) also entered Phase 1 clinical trials (NCT00765973) to test its safety and efficacy. Up till now, these four products have completed their clinical trial phase, but no updated information has been released.

Three products, including LiPlaCis, INX-0076 and TLD-1 have been in progress of clinical trials. INX-0076 was formulated into a liposome with topotecan for advanced solid tumors. LiPlaCis, developed for treatment of advanced solid tumours, is a liposomal formulation, incorporating cisplatin, which is composed of lipids with degradation properties controlled by the sPLA2 enzyme for a tumour-triggered release mechanism [82]. TLD-1 is a novel liposome encapsulating doxorubicin, indicated for advanced solid tumors [83].

## 4. Lipid-Based Nanoparticles for Gene Therapy

**Figure 5:**
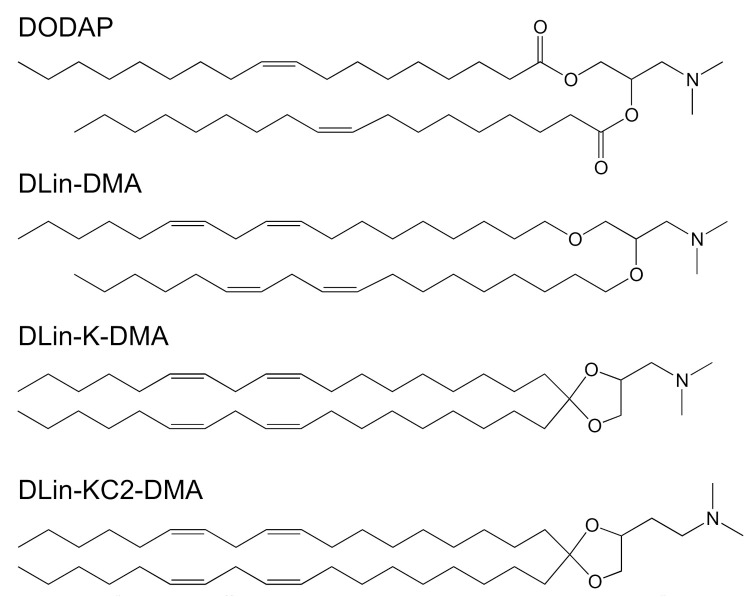
Chemical structures of the most common ionizable cationic lipids: 1,2-dioleoyl-3-dimethylaminopropane (DODAP), 1,2-dilinoleoyl-3-dimethylaminopropane, 1,2- dilinoleyloxy-3-dimethylaminopropane (DLin-DMA), 2,2-dilinoleyl- 4-dimethylaminomethyl-[1,3]-dioxolane (DLin-K-DMA) and 2,2-dilinoleyl-4-(2- dimethylaminoethyl)-[1,3]-dioxolane (DLin-KC2-DMA).

Recently, nucleic acid therapeutics, such as small interfering RNAs (siRNA), small activating RNAs (saRNA), and messenger RNA (mRNA), have gained much traction and have been at the forefront of medicine with their potential in delivery efficiency and treatment of a wide range of diseases [87,88]. However, these genetic drugs are prone to rapid degradation by serum endonucleases. To protect them, liposomes or lipid nanoparticles (LNPs, including SLNs and/or NLCs) have been increasingly utilized as a delivery system which have sufficient encapsulating capacity and which are capable of targeting tissues and cells [89]. A series of ionizable lipids have been designed for gene delivery, each with their own pKa and structural properties, such as 1,2-dioleoyl-3-dimethylaminopropane (DODAP, pKa of 6.6), 1,2-dilinoleoyl-3-dimethylaminopropane, 1,2- dilinoleyloxy-3-dimethylaminopropane (DLin-DMA, pKa of 6.8), 2,2-dilinoleyl- 4-dimethylaminomethyl-[1,3]-dioxolane (DLin-K-DMA, pKa of 5.94), 2,2-dilinoleyl-4-(2- dimethylaminoethyl)-[1,3]-dioxolane (DLin-KC2-DMA, pKa of 6.68) (Figure 5) [18], and (6Z,9Z,28Z,31Z)-heptatriaconta-6,9,28,31-tetraen-19-yl-4-(dimethylamino)-butanoate (Dlin-MC3-DMA, pKa of 6.44) (Figure 1). Aside from the pKa value, the linker between the head group and alkyl chains is also a determinant for delivery efficiency. The ketal linker was demonstrated to be the best candidates when compared to ester- and alkoxy-linkers [18]. Dlin-K-DMA with significant in vivo silencing of factor VII was compared to other lipids without ketal linkers [18]. A series of linker optimization, resulted in DLin-MC3-DMA, which is now considered as a promising candidate for genetic drug delivery and able to launch in the clinic. Dlin-MC3-DMA exhibited a 10-fold higher potency than Dlin-KC2-DMA for hepatic gene silencing in vivo [21]. An illustration of this lipid’s potency is provided with Onpattro^®^ (Figure 1). The second siRNA therapeutic to receive FDA approval was GIVLAARI^TM^ (givosiran, ALN-AS1) in November 2019 [90] and a market authorization from the European Committee—a synthetic siRNA targeting the ALAS1 gene in hepatocytes. This therapy is prescribed for adult patients with acute hepatic porphyria, a genetic disorder resulting in the buildup of toxic porphyrin molecules which are formed during the production of heme. This RNA therapy, however, is composed naked nucleotide-modified siRNA without a carrier, but in the presence of a targeting ligand that directs the drug towards the liver.

An LNP platform developed by Arcturus Therapeutics, Inc. (San Diego, CA, USA) for RNA delivery was named as lipid-enabled and unlocked nucleomonomer agent modified RNA (LUNAR^®^). LUNAR is made of a proprietary ionizable amino lipids (58 molar %, ATX, Figure 4), a phospholipid 1,2-distearoyl-sn-glycero-3-phosphocholine (7 molar %, DSPC), cholesterol (33.5 molar %) and DMG-PEG_2000_ (1.5 molar %) [91]. The ATX lipids are similar to a lipid family developed by Alnylam/Acuitas that can be modified to target specific cells type or tissues for a variety of indications. Unlike the conventional cationic lipids, the ATX lipids are degraded under physiological conditions through the breaking of the ester linkages, thereby facilitating rapid degradation for faster metabolism and better safety profile [92]. It was shown that LUNAR was employed to encapsulate the human FIX (hFIX) mRNA for treatment of hemophilia B in a preclinical setting [93]. The efficacy of LUNAR in mRNA delivery was 5-fold higher than other lipid carriers with DLin-MC3-DMA or heptatriaconta-6,9,28,31-tetraen-19-yl-4-(dimethylamino)butanoate) (MC3). Additionally, the LUNAR formulation did not elicit an adverse immune response, such as an increase of liver enzymes that are markers for acute liver toxicity [93]. 

Aside from Onpattro as the first commercial RNA product, GIVLAARI^TM^ and mRNA-LUNAR in preclinical studies, a variety of RNA-LNP have now entered clinical trials (Table 4). TKM-080301 was studied and produced by Arbutus Biopharma Corporation for the treatment of solid tumors, such as gastrointestinal neuroendocrine tumors or adrenocortical carcinoma (ACC) [94]. TKM-080301 is composed of a siRNA encapsulated in LNPs that can target polo-like kinase 1 (PLK1), which regulates critical aspects of tumor progression. A phase I/II clinical study was conducted (NCT01262235) with promising safety and anti-tumor efficacy data [94,95]. After testing on 16 patients at 0.6 or 0.75 mg/kg/week for 18 cycles, it was confirmed that TKM-080301 could increase PLK1 expression and inactivate the target in ACC. Later, in 2018, TKM-080301 was clinically evaluated for safety, pharmacokinetics and preliminary anti-tumor activity in patients with advanced hepatocellular carcinoma (NCT02191878) [96,97]. In this early-phase study with 43 patients, the antitumor effect of TKM-080301 was limited. Consequently, TKM-080301 did not continue for further evaluation as a single agent for the treatment of HCC, and clinical studies have been terminated [97].

Dicerna Pharmaceuticals (Lexington, Massachusetts MA, USA), a company specializing in RNA medicines that silence genes has developed DCR-MYC, a lipid particle that incorporates synthetic double-stranded RNA to target the MYC oncogene and suppress cancer progression [26,98]. This therapy has been evaluated in a dose escalation study in patients with multiple myeloma, lymphoma or solid tumors (NCT02110563). Additionally, DCR-MYC was also evaluated in clinical trials for hepatocellular carcinoma (NCT02314052) [99]. However, all clinical studies related to DCR-MYC have been stopped by Dicerna, as the early efficacy results did not meet the company’s expectations to warrant further development [99,100].

In the line of using genetic therapies for cancer applications, Wagner et al. developed a nanoliposomal EphA2-targeted therapeutic (EphA2 siRNA), which has a neutral charge and is aimed at reducing organ toxicity associated with charged particles [101]. EphA2 belongs to a subfamily of the tyrosine kinase receptors, and is overexpressed in breast, lung, prostate, ovarian, pancreatic, and endometrial cancer [101]. The encapsulated siRNA therefore disturbs the cancer cell proliferation and slows down tumor growth. In preclinical studies, this formulation exhibited a significant anti-cancer effect with mild responses of hemolytic reaction, inflammation and mononuclear cell infiltration in gastrointestinal tract, heart and kidney [102]. These promising results have led EphA2 siRNA to entering phase 1 clinical trial (NCT01591356), where it will be used in patients with advanced or recurrent solid tumors [103].

ModernaTX, Inc. and AstraZeneca have developed the mRNA-2752 encapsulated LNP and have applied for a phase 1 clinical trial (NCT03739931). This mRNA encodes for OX40L, a T-cell co-stimulator, IL-23 and IL-36γ pro-inflammatory cytokines. Patients with solid tumors were treated individually with mRNA-2752 or in combination with durvalumab. Tumor shrinkage in monotherapy or dual-therapy with durvalumab was observed in pre-clinical studies in patients with advanced solid malignancy or lymphoma, which supports the move of mRNA-2752 towards phase 1 studies. Using similar technology, ModernaTX, Inc. studied another LNP candidate (mRNA 2416) for treatment of patients with advanced malignancies [110]. mRNA-2416 is delivered directly to tumors that over-express OX40 which then activate strongly T cell responses that kill the tumor. Currently, this candidate has been recruiting for phase 1–2 clinical trials with identifier of NCT03323398 [110].

Alnylam Pharmaceuticals also completed a Phase 1 clinical trial (NCT01158079) for ALN-VSP02 in 2012 [107], which is an LNP with siRNA that targets the expression of vascular endothelial growth factor (VEGF) and kinesin spindle protein (KSP) [106]. These factors are overexpressed in many tumors and contribute to tumor proliferation and survival. Therefore, ALN-VSP02 silences these two mRNAs and prevents the translation of the KSP and VEGF proteins, which inhibits tumor growth in these patients. 

The LNP-RNA system has proven to be a versatile platform with uses beyond cancer treatments. From 2009 to 2019, there were 6 similar products approved for clinical trials (Table 4). Arbutus Biopharma Corporation completed phase 2 clinical trials (NCT02631096) for ARB-001467 [17]. This formula contained siRNAs against the four hepatitis B virus transcripts and was indicated for patients with hepatitis B. Bristol-Myers Squibb and Nitto Denko Corporation completed a phase 1b/2 clinical trial of ND-L02-s0201, which was indicated for patients with moderate to extensive hepatic fibrosis (NCT02227459). ND-L02-s0201, a heat shock protein 47 siRNA, moderates collagen synthesis and secretion to prevent the fibrosis. This clinical study has started in Japan after being initiated in Europe and the United States. Arrowhead Research had also generated the ARC-520 to treat hepatitis B virus and applied phase 2 clinical trials (NCT02065336). ARC-520 formulated from interference RNA, which can reduce all RNA transcripts derived from covalently closed circular DNA that leads to reduce viral antigens and hepatitis B virus DNA. However, ARC-520 was terminated due to delivery-associated toxicity [112]. Currently, Dicerna Pharmaceuticals, Inc. has developed LNPs with siRNA (named as DCR-HBVS) targeting the mRNA for the hepatitis B surface antigen (HbsAg) for treatment of chronic hepatitis B. Phase 1 clinical trial for DCR-HBVS has been requested (NCT03772249) when promising preclinical data was obtained, in which a mouse model of hepatitis B, treated by DCR-HBVS was significantly reduced.

Alnylam Pharmaceuticals also completed a phase 1 clinical trial (NCT01437059) for ALN-PCS02 for the treatment of patients with hypercholesterolemia. ALN-PCS02 is formulated from siRNA encapsulated in lipid LNPs, in which siRNA reduces the proprotein convertase subtilisin/kexin type 9 (PCSK9) enzyme of plasma cholesterol metabolism leading to lower levels of low-density lipoprotein [102].

Tekmira registered a phase 1 clinical trial with identifier of NCT00927459 for PRO-040201 in 2009, which contains siRNA loaded in a stable nucleic acid LNP. PRO-040201 can target ApoB produced by hepatocytes to control the level of cholesterol in blood. Although the preliminary clinical trial demonstrated that PRO-040201 delivered siRNA effectively to liver and reduce significantly low density lipoprotein, there were flu-like symptoms at the highest dose [102]. Thus, Tekmira decided to terminate the study in 2010.

## 5. Lipid-Based Nanoparticles for Vaccines

At the ending of 2019, Wuhan city, Hubei province of China appeared pneumonia patients. After that, in January 2020, a novel coronavirus causing pneumonia was confirmed by Chinese under the name of 2019 nCoV. World Health Organization (WHO), Chinese authorities and other partners have worked to understanding about properties, sources, prevention of virus spread, and treatments [113]. However, COVID-19 pandemic is spreading all over the world unprecedentedly that impacts on global economy individual and community health seriously. As a result, urgent demand is not only to save COVID-19 patients but also to develop vaccines.

Nucleic acid-based vaccines have gained much attention and the first candidates have entered clinical trials [114]. Nucleic acid vaccines possess many advantages over conventional protein-based vaccines such as ease of synthesis, safety, effective antigen manipulation, cost, and scale-up ability [115,116]. However, nucleic acids display some inherent disadvantages [114,116]. DNA has low immunogenicity and might integrate with human genome [116]. RNA is rapidly degraded in physiological media and efficiently excreted by glomerular filtration within less than 10 min [14]. In spite of that, RNA is still considered the best platform technology for developing vaccines against various diseases, both non-infectious diseases and infectious [117]. In vitro transcribed RNA vaccines exhibit efficient antigen expression and self-adjuvancy [118]. Adjuvants are often added to vaccines in order to enhance and prolong the immune response [119], but they also have the potential to cause inflammatory side effects [120]. Therefore, RNA vaccines are becoming a promising candidate as self-adjuvant vaccines with minimal inflammatory side effects [121]. Particles for vaccine delivery have to achieve a high loading capacity, sustainable release, no leakage, and simple manufacturing [122]. To maximize the efficacy of those vaccine candidates as well as to achieve a favourable index for human applications, scaffolds carrying those agents play an important role in vaccine efficacy. Careful consideration of appropriate scaffolds for a specific vaccine plays a key role in vaccine fabrication. Related to liposome scaffolds, four other approved liposomes Inflexal V, Epaxal, Mosquirix and Shingrix were commercialized successfully as vaccines [6,7,71]. Those liposomes are incorporating virosomal influenza vaccine, inactivated hepatitis A virus, RTS,S antigen-based vaccine, and glycoprotein E based vaccine [6,7]. LNPs have become ideal candidates for vaccine design, they are similar to viral structures with virus-like dimensions, and able to carry antigens and adjuvants [123]. 

Along with the history of vaccine development, adjuvants are equally as important and have led to significant advances in vaccine formulations and efficacy thereof. Especially Alum (insoluble aluminium salts) is incorporated in many childhood vaccines such as DTaP (diphtheria, tetanus, pertussis) vaccines, the pneumococcal conjugate vaccine, and hepatitis B vaccines [124,125]. Of other approved vaccines in 2015, there were many formulations that included adjuvants. For example, AS01 (MPL (a naturally derived TLR4 ligand)and QS21 saponin), MF59, and immunostimulatory oligonucleotides are presented in Shingrix, Fluad and Heplisav, respectively [124]. Besides, various other vaccine adjuvants including AS04, RC-529, CpG ODN, TLR9 agonist, TLR4 agonist, and virosomes have been included in licensed products [124,125]. Those vaccines confirm that incorporation of adjuvants can progress the vaccine into the clinic more rapidly and successfully. Also, a broad range of lipids has been reported to possess the strong adjuvant activity. Especially, cationic lipid, dimethyldioctadecylammonium bromide (DDA), showed the deposition of the antigen at the injection site, the enhancement of a cellular antigen internalization, and an antigen association [126]. As a result, LNPs made from DDA were forecasted to possess self-adjuvant activity. Indeed, Anderluzzi et al. reported that an emulsion of polymeric nanoparticles, liposomes, and solid lipid nanoparticles structured by DDA gained high antigen adsorption efficiency, in vitro antigen trafficking, in vivo distribution and high antibody response [126]. However, the immunogenicity level was strongly dependent on the type of formulation. The nanoparticle system exhibited high cell uptake and antigen processing, while the emulsion showed high antibody responses [126]. 

In another case, LNPs were used as a carrier system for adjuvants and mRNA to achieve their synergistic effects in immune stimulation. Lee et al. fabricated the LNPs with an adjuvant of Pam3 (tri-palmitoyl-S-glyceryl cysteine linked to the penta- peptide) to carry mRNA for cancer immunotherapy. Their results indicated that this formulation triggered different TLR’s to increase the population of CD8+ T cells, thereby preventing tumor growth. So the combination of an adjuvant and mRNA in an LNP carrier could be a promising avenue in mRNA-based cancer therapeutics [118].

### 5.1. Lipid-Based Nanoparticles for Therapeutic Vaccines

**Figure 6:**
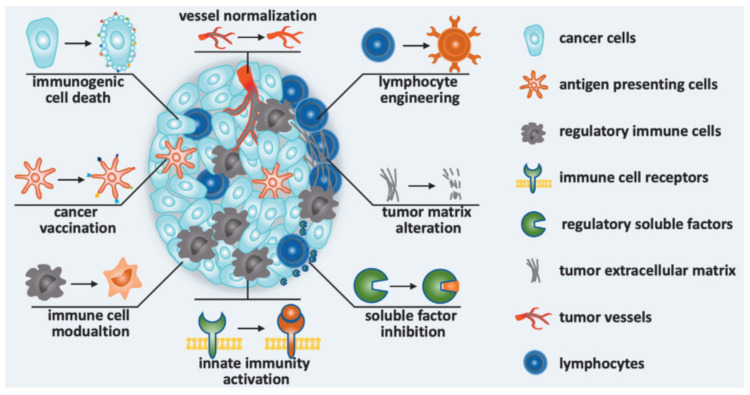
Immunomodulation strategies to improve cancer immunotherapy in nanomedicines: Nanomedicine was designed to induce immunogenic cell death, to promote antitumor immunity (cancer vaccination), to modulate immune cells, to activate innate immunity, to inhibit soluble immunosuppressive factors, to alternate tumor matrix, to engineer lymphocyte and normalize vessel [129].

LNPs and liposomes showed their best suitability for RNA-based vaccines in protective ability, pharmacokinetics, tissue distribution and targeted delivery (dendritic cells and macrophages) [12,114,122,127,128]. With optimization of over 1000 candidates, it was concluded that LNPs are suitable delivery vehicles for mRNA coding antigens for anti-cancerous vaccines [13]. Such an RNA-LNPs have been designed to reverse M2-like macrophages or other immunosuppressive phenotypes, to activate the innate immunity, to inhibit other soluble immunosuppressive factors, and to induce tumor-infiltrating lymphocytes for immunomodulation strategies to treat malignant tumors (Figure 6). In fact, there are a lot of studies related to this topic that have been summarized in the selected reviews [12,13,14]. Herein, we will focus on the clinical trials of developed vaccines, summarized in Table 5.

Lipo-MERIT is a cancer vaccine [133] that is made of four mRNA’s encoding for NY-ESO-1, MAGE-A3, tyrosinase and TPTE which are encapsulated in liposomes without any modification with molecular ligands [134]. Lipo-MERIT travels to spleen and is taken up by splenic dendritic cells and macrophages to activate NK, B, CD4^+^, and CD8^+^ T cells. For the progress of commercial preparation, Lipo-MERIT is currently in Phase 1 clinical trials [130].

Another cancer vaccine in clinical trials is the mRNA-LNP personalized cancer vaccine, mRNA-4157, developed by ModernaTX, Inc. and Merck Sharp & Dohme Corp. This vaccine is indicated for patients with resected solid tumors including bladder carcinoma, melanoma and non-small cell lung carcinoma (NSCLC). In addition, mRNA-4157 is also used in combination with pembrolizumab for patients with advanced or metastatic cancers. After administration, this LNP is uptaken and translated by antigen presenting cells, thereby inducing both cytotoxic T-lymphocyte and memory T-cell-dependent immune responses to destroy the cancer cells. Currently, mRNA-4157 is being clinically evaluated for safety and efficacy in Phase 1 (NCT03313778) [131] and Phase 2 (NCT03897881) [132].

Stimuvax (L-BLP-25, BLP25 liposome) of EMD Serono & Merck KgaA, Darmstadt, Germany was also entered phase 3 clinical trial (NCT00409188). This therapeutic vaccine provides immunity to kill the cancer cells expressing a glycoprotein antigen of Mucin 1 (MUC-1). The trial for patients with advanced non-small cell lung cancer (NSCLC), but the results were disappointing and did not meet the primary endpoint [135]. As a result, L-BLP-25 was terminated.

### 5.2. Lipid Nanoparticles for Prophylactic Vaccines—A Rapid Response to COVID19:

In March 2020, the World Health Organization (WHO) declared that the Coronavirus Infectious Disease (COVID-19) was a global pandemic. In March 2021, more than 129.4 million cases and 2.8 million deaths were reported all over the world [123]. Concurrently, COVID-19 has a high probability of becoming a seasonal disease with high infection rates and a long incubation period [123]. Consequently, developing COVID-19 vaccines has been a necessity for the global population [136], for which numerous platforms have been investigated. There are 12 vaccines approved by the FDA. Others are in clinical trials, 93 vaccines are listed with 257 trials [127,137], of which, 29 vaccines in Phase 1, 39 vaccines in Phase 2, and 25 vaccines in Phase 3 [137]. The activating mechanisms in most of those vaccine candidates are based on the induction of neutralizing antibodies against the spike (S) protein to prevent the uptake into human cells via the human angiotensin-converting enzyme-2 (ACE2) receptor [127]. Thus far, for mRNA-LNPs only mRNA-1273 and BNT162 have been successfully developed and others are progressing in clinical trials (Table 6) [12,128,138,139,140].

LNPs encapsulating mRNA developed by BioNTech SE and Pfizer for the SARS-CoV-2 vaccine had four candidates (BNT162a1, BNT162b1, BNT162b2, and BNT162c2). They were designed from two types of a nucleoside-modified mRNA, a uridine containing mRNA and a self-amplifying RNA [138,141]. They have been tested in Phase 2 clinical trials (NCT04380701)in healthy volunteers aged from 18 years to 85 years [142], and in Phase 3 (NCT04368728) [143]. The BNT162b2 candidate has finished phase 3 clinical trials with promising results of a safe and effective vaccine. In front of the urgent need of Covid-19 vaccine, Pfizer and BioNTech submitted their BNT162b2 to the FDA to request an emergency use authorization. On 11 December 2020, the FDA approved Pfizer-BioNTech COVID-19 Vaccine distributed in the United States [144].

Using a similar strategy, mRNA-1273 COVID-19 is fabricated from synthetic mRNA inside LNPs [128,145]. This synthetic mRNA encodes for the spike, S protein of SARS-CoV-2 viruses [12,138,139] which is a key factor on viral surfaces binding to the host cell through ACE2 receptor. The S protein of SARS-CoV-2 viruses mediates cell attachment, receptor recognition, and fusion for viral penetration and infection [146]. This formula has been developed by Moderna TX, Inc, and in the process of FDA approval (phase 1) with reference number of NCT04283461 [127,128,147]. It also moved to phase 2 (NCT04405076) to be assessed for reactogenicity, immunogenicity and safety in healthy male and non-pregnant females from 18 years old [140,148]. This candidate has entered Phase 3 (NCT04470427) to be evaluated for safety, efficacy and immunogenicity to prevent COVID-19 for up to two years [140,149]. On 17 December 2020, Moderna TX, Inc gained the emergency-use authorization of the FDA for mRNA-1273 [150].

McKay et al. studied LNPs encapsulated with self-amplifying RNA (saRNA) as the new SARS-CoV-2 vaccine named LNP-nCoVsaRNA or COVAC1 [136,151]. These self-amplifying RNA constructs have been proposed because any antigen of interest can be encoded and formulated at a lower dose than conventional mRNA. For in vitro and in vivo experiments, the LNP-nCoVsaRNA vaccine for SARS-CoV2 exhibited robust antibody and cellular responses. These outstanding results induced the strong belief that the LNP-nCoVsaRNA vaccine would promote immunogenicity in humans. Currently, Imperial College London applied the LNP-nCoVsaRNA vaccine for the clinical trial, currently in Phase 1 (ISRCTN17072692) [140,152].

Duke-NUS Medical School and Arcturus Therapeutics Inc. have collaborated for the development of a COVID-19 vaccine named ARCT-021. They used the LUNAR lipid-mediated delivery system to encapsulate RNA (STARR^TM^). In the preclinical stage, ARCT-021 has demonstrated to be a safe and efficient vaccine for COVID-19 at low dose. It was able to induce CD8+ T-cell and T-helper cellular immune responses without adjuvants and viral vector. Moving to Phase 1/2 clinical studies (NCT4480957), ARCT-021 has been evaluated with safety, tolerability and immunogenicity at multiple dose levels from 1–10 μg. The age groups for this study are healthy people from 18–80 years, with the exception of pregnant and breast-feeding women. Preliminary data for ARCT-021 showed a favourable safety profile at a relatively low dose [153]. In addition, other similar vaccine platforms including ChulaCov19 mRNA vaccine (Chulalongkorn University) and SARS-CoV-2 mRNA vaccine (Shulan, HangzhouHospital) have applied for clinical trials. The ChulaCov19 mRNA vaccine has been registered for phase 1 clinical trials (NCT04566276) with no study recruitment yet [151]. The SARS-CoV-2 mRNA vaccine was applied to Chinese Clinical Trial Registry for Phase 1 clinical trial (ChiCTR2000034112). This trial will evaluate the safety, tolerance and immunogenicity of multiple doses in the population above 18 year olds [151].

The LNPs under development for the COVID-19 vaccines do not only include the aforementioned approved products or those still in the clinical trials, but also include numerous others that are continuously being developed in pre-clinical stage (Table 6) [154]. Many companies, research institutes, and universities worldwide have been listed on the WHO list with RNA-encapsulated LNP vaccines. Globe Biotech Ltd. had SARS-CoV-2 D614G variant LNP-encapsulated mRNA. Max-Planck-Institute of Colloids and Interfaces had LNPs encapsulating mRNA targeting Langerhans cell. Sanofi Pasteur and Translate Bio studied the mRNA-based vaccine MRT5500 and are going to apply for clinical trial at the ending of 2020 [155]. Now, MRT5500 has started phase1/2 clinical trial. It was expected to get interim results in the third quarter of 2021 [156]. CanSino Biologics and Precision NanoSystems had a collaboration to develop a COVID-19 RNA vaccine composed of mRNA and lipid nanoparticle carrier [157]. Daiichi-Sankyo Co. has developed mRNA-based COVID-19 vaccine named as DS-5670. For next clinical studies, Daiichi-Sankyo Co. is going to collaborate with the University of Tokyo [140]. IMV Inc (Dartmouth, Canada), formulated their DPX-COVID-19 vaccine candidate. DPX is the lipid-based delivery system in which peptide antigens are dissolved in lipids with the final formulation stored in dry form [158]. After dissolution, this vaccine is injected intramuscularly. There is no releasing mechanism at the injection site, but the peptide antigens act as adjuvant and the formulated DPX can then recruit the antigen presenting cells that induce an immune response from the lymph nodes [158]. Na-Na Zhang et al. studied a thermostable mRNA vaccine candidate for preventing COVID-19 infection [159]. They used LNPs to encapsulate mRNA targeting the receptor-binding domain (RBD) of the severe acute respiratory syndrome coronavirus 2 (SARS-CoV-2). In the mouse model they used, the this LNP induced neutralizing antibodies and T-cell responses with high protective immunity against SARS-CoV-2. Additionally, this vaccine candidate can be stored at room temperature for at least one week. With these advantages, this LNP-mRNA candidate (RQ3011-RBD) is moving into phase 1 clinical trial evaluation by Fudan University, Shanghai JiaoTong University and RNACure Biopharma [140,159]. Moreover, they designed two other vaccine candidates, RQ3013-VLP and RQ3012-Spike, that include a cocktail of mRNA constructs. The RQ3013-VLP candidate contains a cocktail of mRNAs encoding 3 viral structural proteins of S (spike), M (membrane), and E (envelope). The RQ3012-Spike vaccine carries mRNA encoding the full-length wild-type S. In mice model, RQ3013-VLP exhibited the best immune response, across three these candidates, while RQ3011-RBD induced insufficient immunity at a low concentration of 2 μg RNA/dose. Depending on these preliminary tests, it can be concluded that mRNA vaccines can act as a flexible platform to design effective candidates [159].

Besides, the developing strategy of vaccines has still studied for other diseases. Shirai et al. demonstrated that LNPs could act as an adjuvant for influenza vaccines [162]. This was confirmed through the results about the immune-stimulatory effects on dendritic cells in mice and the protection ability of LNPs encapsulating the conventional seasonal split vaccine (SV) in comparison with bare SVs and SVs combined Alum. The LNP was made of 1,2-dioleoyl-3-trimethylammonium- propane, 1,2-dipalmitoyl-sn-glycero-3-phosphocholine, N-(carbonyl-methoxypolyethyleneglycol 2000)-1,2-distearoyl-sn-glycero-3-phosphoethanolamine and cholesterol. It was found that the LNPs with SVs achieved a similar efficiency as SVs combined Alum. Alum induced a high inflammatory response, which is considered a limitation of traditional adjuvants. In contrary, LNPs were able to induce SV-specific immune responses without inflammation [162]. Swaminathan et al. also assessed the adjuvant activity of LNPs alone and of LNPs incorporating the synthetic TLR9 agonist, IMO-2125 adjuvant, in a mouse model [163]. This LNP formulation was composed of DSPC, cholesterol, DMG-PEG2000 and an asymmetric ionizable amino lipid. Surprisingly, the LNP without adjuvant was able to induce B-cell responses against HbsAg (hepatitis B virus surface antigen) and ovalbumin sub-unit antigens at a comparable level than in the presence of other adjuvants including IMO-2125, 3-O-deactytaled monophosphoryl lipid and aluminum-based adjuvants. The LNP not only induced a significant enhancement of immune responses but also elicited a higher Th1-type response compared to IMO-2125 alone. So the combination of LNPs and immune-modulatory oligonucleotide adjuvants led to have the synergistic effects for immune responses and to manipulate those immune qualities that is quite different from the inorganic adjuvants.

## 6. Conclusions and Future Directions

The clinical development of lipid-based nanoparticle technologies with chemo- and nucleic acid therapeutics have demonstrated the potential of lipid-based carriers in the treatment of a range of diseases. However, the number of successful products that have reached the market does not accurately represent the number of formulations in (pre)clinical trials, indicating that the development of these nanoparticles still suffers from difficulties and challenges in the translation from animals to humans. Recently, several strategies have been developed to overcome these limitations. To improve the stability of nanoparticles and prevent drug leakage, lipid structures have been designed that efficiently complex by ionic attraction with the encapsulated therapeutic. Ionizable lipids, such as DOP-DEDA, have shown to be favourable for gene encapsulation. Cholesterol, on the other hand, is essential in providing stability to the liposomal structures resulting in tight packing of the drugs. Stability of the LNPs in physiological media and systemic circulation is achieved by modifying the particle surface with a PEG-lipid, thereby reducing the recognization by the reticuloendothelial system. However, the production of anti-PEG antibodies following administration of the first dose has been reported to reduce the therapeutic efficacy and/or cause adverse reactions upon the following doses. The quest for PEG alternatives has, therefore, become necessary to enable repeated injections. Besides the prolonged circulation, LNPs must target the specific tissues/cells/organs and then internalize through cell membranes to release drugs at the site of action. To achieve this requirement, LNPs have been designed as smart materials with selective ligands with degradation being triggered by changes in pH, temperature or oxidation/reduction. 

The selective association of LNPs with target cells remains a challenge. Nucleic acid vaccines for example cannot be injected directly into our lymph nodes or spleen, which are tissues that are home to immune cells responsible for making antibodies and killing cancer cells. The targeted delivery of nucleic acids from injection sites to immune cells in lymph nodes or the spleen is therefore critical to maximising the production of antibody or long-lived antigen-specific cytotoxic T cells. Such delivery systems need the ability to target lymph nodes but also cross the barrier cells in the lymph nodes to interact with immune cells. Additionally, after internalization, the controlled release of therapeutics to aberrant cells has to be initiated effectively. These mechanisms that facilitate cell uptake, internalization, and payload release have not yet achieved the expected results. The success of LNPs with selective ligands in the market have not been completed yet. Therefore, with various cell type-specific ligands and stimulus agents, it is expected that studies related efficacy of modified LNPs for different diseases will enter clinical trials soon. The co-delivery or drug co-encapsulating with adjuvants are of interest in this field to improve efficacy and immune modulation. Additionally, the manufacturing and scaling up process of LNPs, has been challenging. New methodologies of LNP preparation based on microfluidics have been considered as the most robust to date, but it exhibits limitations in formulating multifunctional LNPs. 

Concurrently, to combat the COVID-19 pandemic, the fast development of RNA and LNP-based vaccines have gained emergency FDA approval, which demonstrates the rapid and effective response of this approach against complex diseases. These vaccines are not limited to infectious diseases but have also been developed for other disease types such as cancer and hyperlipidemia. Some clinical trials are almost at the final phase. Numerous studies are at early stages and will increase in the future, which forecasts a range of products to be launched on the market.

In conclusion, we believe that the success of mRNA-LNP vaccines opens an exciting chapter for LNP technology. A long road to optimizing LNP formulations for small molecule drugs and nucleic acid delivery has been paid off, and LNPs have become, once again, a frontrunner in nano drug delivery system. This review highlights key lessons learnt from this long road and serves as a reference for designing LNPs. Further development of LNPs is still urgently needed to address current global health challenges, which requires collaborative efforts of scientists in different fields.

## Figures and Tables

**Table 1:** Summary of main characteristics of common lipid-based nanoparticles.

Particle Type	Composition	Shape/ Size	Preparation	Advantages	Drawbacks
Liposomes	Phospholipid, cholesterol, essential oils [31].	Spherical, 10–1000 nm	Mechanical dispersion, Solvent dispersion, Detergent removal	Drug protection, controlled release, solubility enhancement for hydrophobic therapeutic agents, high bioavailability and biodistribution	Not crossing the stratum corneum barrier, rigid structure [31]
Niosomes	Cholesterol, non-ionic surfactants [31]	Spherical , 10–1000 nm	Sonication, micro-fluidization, ether injection method, bubble method	Targeting to specific sites, enhanced stability and longer shelf life than liposomes	Drug leakage, particle aggregation [30], high production costs and the scarcity of FDA-approved polymers [29]
Transfersomes	Phospholipids and edge activators [31]	Spherical , <300 nm	Rotary film evaporation, reverse-phase evaporation, vortexing sonication	Higher penetration, good stability	Highly prone to oxidative degradation, high cost and impurity of natural phospholipids
Solid lipid nanoparticles (SLNs)	Solid fats, surfactants [32]	Spherical, 50–1000 nm	Micro emulsification, sonication, high pressure homogenization [27,33]	Biocompatible and biodegradable ingredients, high cell uptake, good protection of drugs in acidic pH, long shelf life, ease of drug entrapment [32]	Gelling tendency [29]
Nanostructured lipid carriers (NLCs)	Solid and liquid lipids (fats and oils), surfactants [32]			All SLN’s advantages but higher drug encapsulation, more sustainable drug release, better diuretic activity and fewer drug lost within storage time [32]	Optimization required of the ratio of solid/liquid lipids

**Table 2:** Overview of the approved liposomes in EU and US.

Initial Approval Time	Commercial Name	Drug	Liposome Components	Treatment
1995	Doxil^®^	Doxorubicin	HSPC: cholesterol: DSPE-PEG [6]	Breast neoplasms; multiple myeloma; ovarian neoplasms; Kaposi’s sarcoma
1995	Abelcet^®^	Amphotericin B	DMPC:DMPG [6]	Life-threatening fungal infections.
1996	DaunoXome^®^	Daunorubicin	DSPC: Cholesterol [6]	Cancer advanced HIV-associated Kaposi’s sarcoma
1997	AmBisome^®^	Amphotericin B	HSPC:DSPG, cholesterol [6]	Visceral leishmaniasis
1999	DepoCyt^®^	Cytarabine	DOPC:DPPG [6]	Neoplastic meningitis
1999	Curosurf^®^	SP-B and SP-C	A natural surfactant of porcine lungs [6]	RDS in premature infants
2000	AmBisome^®^	Amphotericin B	HSPC:DSPG, cholesterol [6]	Cryptococcal Meningitis in AIDS Patients
2000	Myocet^®^	Doxorubicin	EPC: Cholesterol	Breast neoplasms
2000	Visudyne^®^	Verteporfin	EPG:DMPC [6]	Sub foveal choroidal neovascularization
2004	DepoDur^®^	Morphine	DOPC:DPPG [6]	Pain relief
2009	Marqibo^®^	Vincristine	SPH: Cholesterol [6]	Philadelphia chromosome-negative acute lymphoblastic leukemia [6]
2009	Mepact^®^	Mifamurtide	DOPC:DOPS [6]	Osteosarcoma
2011	Exparel^®^	Bupivacaine	DEPC: DPPG: Cholesterol: Tricaprylin [6]	Anesthetic
2012	Marqibo^®^	Vincristine	SPH: Cholesterol [6]	Hematologic malignancies and solid tumors [7,71]
2013	Lipodox^®^	Doxorubicin	HSPC: Cholesterol: DSPE-PEG [6]	Breast neoplasms
2015	Onivyde^®^	Irinotecan	DSPC: Cholesterol: DSPE-PEG [6]	Metastatic pancreatic cancer
2017	Liposomal doxorubicin	Doxorubicin	HSPC: Cholesterol: DSPE-PEG [6]	Breast neoplasms
2017	Nocita^®^	Bupivacaine	DEPC: DPPG: Cholesterol: Tricaprylin [6]	Anesthetic
2017	Vyxeos^®^ (CPX-351)	Daunorubicin Cytarabine	DSPC: DSPG: Cholesterol [6]	Acute myeloid leukemia
2018	ArikayceTM	Amikacin	DPPC: Cholesterol [6]	Mycobacterium avium complex lung disease
2018	LipoplatinTM NanoplatinTM	Cisplatin	DPPG: soy PC: MPEG-DSPE: Cholesterol [72]	Pancreatic cancer Lung cancer

**Table 3:** Liposomal formulations in clinical trials.

Drug & Sponsor	Drug/Target	Clinical Trial	Indication	Ref.
Liposome Encapsulated Mitoxantrone (LEM) INSYS Therapeutics Inc (Phoenix, AZ, USA)	Mitoxantrone	Phase 1 NCT00024492 Completed	Tumors	[72]
S-CKD602 University of Pittsburgh ALZA	CKD-602	Phase 1 NCT00177281 Completed	Advanced Malignancies	[82]
Topotecan Liposomes Injection (TLI) Spectrum Pharmaceuticals, Inc (Henderson, NV, USA)	Topotecan	Phase 1 NCT00765973 Completed	Small Cell Lung Cancer Ovarian Cancer Solid Tumors	[84]
INX-0076 Inex Pharmaceuticals	Topotecan	Phase 1	Advanced solid tumours	[82]
TLD-1 Swiss group for clinical cancer research	Doxorubicin	Phase 1 Recruiting NCT03387917	Advanced Solid Tumors	[83]
LEP-ETU INSYS Therapeutics Inc (Phoenix, AZ, USA)	Paclitaxel	Phase 1 NCT00080418 Completed Phase 2 NCT01190982 Completed NCT00100139 Completed	Advanced cancer (Neoplasm) Metastatic Breast Cancer	[72,82]
MBP-426^®^ Mebiopharm Co., Ltd.	Oxaliplatin / Transferrin	Phase 1/2 NCT00964080	Solid Tumors	[85]
MM-302 Merrimack	Doxorubicin / Antibody fragment	Phase 1/2 Terminated	Breast cancer	[75]
LiPlaCis Oncology Venture	Cisplatin	Phase 1/2 NCT01861496	Phase 1: Advanced or Refractory Solid Tumours Phase 2: Metastatic Breast Cancer, Prostate Cancer and Skin Cancer	[82]
SPI-77 NYU Langone Health	Cisplatin	Phase 2 NCT00004083 Completed	Ovarian Cancer	[72]
OSI-211 Astellas Pharma Inc, OSI Pharmaceuticals (Farmingdale, NY, USA)	Lurtotecan	Phase 2 NCT00046787 Completed	Recurrent Small Cell Lung Cancer	[72,82]
ThermoDox^®^ Celsion	Doxorubicin / Targeted thermal therapy	Phase 3 NCT02112656 completed	Hepatocellular Carcinoma	[86]
MBP-Y003 Mebiopharm Co., Ltd.	Methotrexate / Transferrin	Not yet	Lymphoma	[73]
MBP-Y004 Mebiopharm Co., Ltd.	Docetaxel / Transferrin	Not yet	Lymphoma	[73]
MBP-Y005 Mebiopharm Co., Ltd.	Gemcitabin / Transferrin	Not yet	Lymphoma	[73]

**Table 4:** List of RNA-encapsulated lipid nano-particles (LNPs) with indication and clinical trial information.

Drug and Its Sponsor	Target	Clinical Trial	Indication	Ref.
DCR-MYC Dicerna Pharmaceuticals, Inc.	Oncogene MYC	Terminated, Phase I, II NCT02314052, NCT02110563	Solid tumors, hepatocellular Carcinoma	[26,99]
TKM-080301 Arbutus Biopharma Corporation (Warminster, PA, USA)	PLK1 (polo-like kinase-1)	Completed, Phase I, II NCT01262235, NCT02191878	Gastrointestinal neuroendocrine tumors, adrenocortical carcinoma tumors, advanced hepatocellular carcinoma	[26,94,95,96]
EphA2 siRNA M.D. Anderson Cancer Center National Cancer Institute (NCI)	EphA2	Recruiting, Phase 1 NCT01591356	Advanced Malignant Solid Neoplasm	[26,101,103]
ARB-001467 Arbutus Biopharma Corporation	HBsAg	Completed, Phase II NCT02631096	Hepatitis B, Chronic	[26]
PRO-040201 Arbutus Biopharma Corporation	ApoB	Terminated, Phase I (Potential for immune stimulation to interfere with further dose escalation.) NCT00927459	Hypercholesterolemia	[26]
ALN-PCS02 Alnylam Pharmaceuticals	PCSK9	Completed, Phase I NCT01437059	Elevated LDL-cholesterol	[26]
ND-L02-s0201 Bristol-Myers Sqyubb Pharmaceuticals	HSP47	Completed, Phase I NCT02227459	Hepatic fibrosis	[26]
ARC-520 Arrowhead Pharmaceuticals and ICON Clinical Research	HBsAg	Terminated, Phase 2 NCT02065336	Chronic Hepatitis B Virus	[23]
DCR HBVS Dicerna Pharmaceuticals, Inc.	HBsAg	Recruiting, Phase 1 NCT03772249	Hepatitis B, Chronic	[23,104]
ALN-VSP02 Lipid Nanoparticle	siRNA-KSP	Completed, Phase 1, NCT01158079	Cancer- Solid tumors	[105,106,107]
mRNA-2752 Lipid Nanoparticle Moderna Tx. Inc. (Cambridge, MA, USA) and AstraZeneca (Cambridge, UK)	OX40L T cell	Recruiting, Phase 1 NCT03739931	Cancer- various	[108,109]
mRNA-2416 Lipid Nanoparticle ModernaTX, Inc.	OX40L T cell	Recruiting, Phase 1–2, NCT03323398	Cancer- Solid Tumor,Lymphoma, Ovarian	[110]
Liposomal T4N5 Lotion National Cancer Institute (NCI)	a prokaryotic DNA repair enzyme	Completed Phase 2 NCT00089180	The Recurrence of Nonmelanoma Skin Cancer	[111]

**Table 5:** Clinical trial information of LNPs for cancer vaccines.

Vaccine	Developer	Indication
Lipo-MERIT	BioNTech RNA Pharmaceuticals GmbH Phase 1: NCT02410733 (*) [130]	Cancer (melanoma)
mRNA-4157	ModernaTX, Inc. and Merck Sharp & Dohme Corp. Phase 1: NCT03313778 (*) [131] Phase 2: NCT03897881 (*) [132]	Cancer (bladder carcinoma, melanoma and non-small-cell lung carcinoma (NSCLC))
Stimuvax (L-BLP-25)	EMD Serono & Merck KGaA, Darmstadt, Germany. Phase 3: NCT00409188 (*) Terminated [72]	Non-small-cell lung cancer

(*) ClinicalTrials.gov identifier.

**Table 6:** List of the COVID-19 vaccines using LNPs.

Vaccine	Developer	References
***Approved by FDA***		
mRNA-1273	ModernaTX, Inc. Phase 1: NCT04283461 (*) Phase 2: NCT04405076 (*) Phase 3: NCT04470427 (*)	[127,128,147,150]
BNT162b2	BioNTech SE and Pfizer Phase 1/2: NCT04380701 (*) Phase 2/3: NCT04368728 (*)	[144]
***In clinical evaluation stage***		
BNT162a1	BioNTech SE and Pfizer Phase 1/2: NCT04380701 (*) Phase 2/3: NCT04368728 (*)	[138,141]
BNT162b1		
BNT162c2		
ARCT-021 (mRNA Lunar-Cov19)	Arcturus Therapeutics and Duke-NUS Phase 1/2: NCT04480957	[153]
COVAC1 (LNP-nCoVsaRNA)	Imperial College London Phase 1: ISRCTN17072692 (*)	[140,151,152]
ChulaCov19 mRNA vaccine	Chula Vaccine Research Center/University of Pennsylvania Phase 1: NCT04566276 (*)	[140]
SARS-VoV-2 mRNA vaccine	Shulan (Hangzhou) Hospital; Center for Disease Control and Prevention of Guangxi Zhuang Autonomous Region Phase 1: ChiCTR2000034112 (*)	[160]
D614G variant LNP-encapsulated mRNA	Globe Biotech Ltd.	[140]
***In pre-clinical stage***		
LNP-encapsulated mRNA encoding S	Max-Planck-Institute of Colloids and Interfaces	[140]
LNP-mRNA	Translate Bio/Sanofi Pasteur	[140]
LNP-mRNA	CanSino Biologics/Precision NanoSystems	[140]
LNP-encapsulated mRNA	University of Tokyo/Daiichi-Sankyo	[140]
Peptide antigens formulated in LNP	IMV Inc	[140]
LNP-encapsulated mRNA encoding RBD	Fudan University/ Shanghai JiaoTong University/RNACure Biopharma	[140,159]
LNP-encapsulated mRNA encoding the full-length wild-type (WT) S		[161]
LNP-encapsulated mRNA cocktail encoding VLP		[140,161]

(*) ClinicalTrials.gov indentifier.

## Data Availability

Not applicable.
